# Pulmonization of Liver: The Unexpected Appearance of Lung POCUS Signs in the Abdomen

**DOI:** 10.24908/pocusj.v11i01.19785

**Published:** 2026-04-22

**Authors:** Jessie Huang, Jina Bai, Todd Cutler, Gregory Mints

**Affiliations:** 1 Division of General Internal Medicine, Section of Hospital Medicine, Weill Cornell Medical Center, New York City, NY, USA; 2 Department of Medicine, Division of Hospital Medicine, Kings County Hospital and SUNY Downstate Medical University, Brooklyn, NY, USA

**Keywords:** POCUS, Air in liver, Air bronchograms, Shred sign, Medical education, Point of care ultrasound, Abdominal air

## Abstract

Point of care ultrasound (POCUS) is an increasingly common tool used in patient evaluation. Rapid interpretation of POCUS findings rely on the operator's familiarity with POCUS patterns. However, over-reliance on heuristics can result in misdiagnosis. We describe a case involving a patient who exhibited characteristic lung POCUS in the abdominal region. This case emphasized the importance of anatomic correlation and the limitations of pattern recognition in isolation.

## Case Presentation

A 58-year-old man with locally advanced hepatocellular carcinoma and portal vein thrombosis presented to the emergency department with one day of severe right-sided abdominal pain, hematemesis, and melena. The patient had multiple recent hospitalizations for upper gastrointestinal bleeding and biliary infections due to a duodenal ulcer with contained perforation and fistulization into the biliary tree. On arrival, he was afebrile, hypotensive to 89/73 mmHg, tachycardic to 132 bpm, and had an oxygen saturation of 100% on 2 L supplemental oxygen.

The abdomen was diffusely tender without peritoneal signs, and the lungs were clear to auscultation. Laboratory studies were notable for hemoglobin of 7.6 g/dL (baseline), white blood cell count of 30 × 10^3^/µL with 20% bands and lactic acid of 7 mmol/L. The leading differential diagnoses for the patient's shock were hemorrhagic shock from a gastrointestinal bleed or septic shock from an intra-abdominal source. The patient received 2 L of intravenous fluids and 2 units of packed red blood cells. Intravenous vancomycin, ertapenem and micafungin were also started empirically. The Rapid Ultrasound for Shock and Hypotension (RUSH) exam was performed to assess for the cause of undifferentiated hypotension as the patient was initially too unstable to proceed to computed tomography (CT).

**Figure 1.**
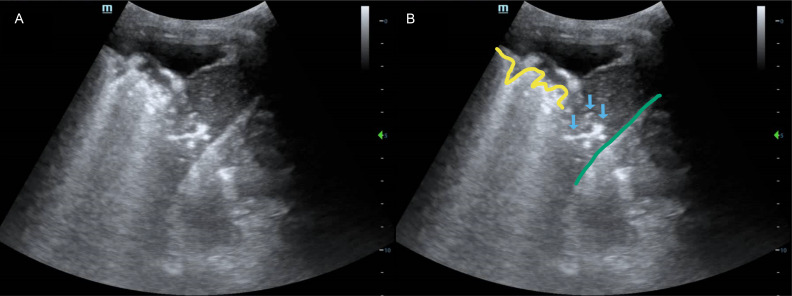
A) Unlabeled point of care ultrasound (POCUS) image of Supplementary Material S1 at 0:00. B) The structures of interest are highlighted. The curved green line highlights what was falsely presumed to be the diaphragm. The blue arrows show hyperechoic foci against a soft tissue density, initially interpreted as “air bronchograms.” The yellow line highlights the irregular hyperechoic line with distal reverberation artifact, initially interpreted as “shred sign.”

The patient was scanned with a curvilinear probe in the supine position. The right flank was interrogated at the posterior axillary line in the coronal plane, with the probe marker oriented cephalad. This showed a curved hyperechoic line at the center of the image, which was initially misinterpreted as the diaphragm (Figure 1, Supplementary Material S1). The liver was not seen caudal to the presumed diaphragm. However, two common POCUS patterns were seen cephalad: (1) several hyperechoic foci on the background of soft tissue density, and (2) an irregular hyperechoic line with distal reverberation artifact. These patterns were recognized as “air bronchograms” and “shred sign” of lung POCUS, respectively. These two signs, when seen in the thorax, indicate the presence of a lung consolidation [1,2]. Although not one of the initial differential diagnoses, septic shock from pneumonia became the leading diagnosis. However, this diagnosis was incorrect.

**Figure 2.**
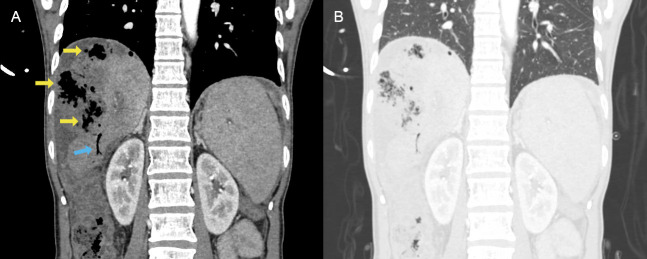
A) A coronal cut of the computed tomography (CT) abdomen. Air is seen within the liver parenchyma in two patterns: large collections of air within the liver masses (yellow arrows), and smaller tracks of air extending to the periphery of the liver (blue arrows). B) Coronal cut of the CT abdomen in the lung preset. The lung bases appear clear.

Subsequent CT angiography of the chest, abdomen, and pelvis demonstrated new focal areas of air within the patient's known liver masses, raising concern for an infected necrotic liver tumor versus hepatic abscesses (Figure 2A). There was also new pneumoperitoneum, suspected to be an extension of the infected liver tumor/abscess. Finally, there were new bilobar branching tracks of air in the liver, which was suggestive of portal venous gas (PVG). No pulmonary consolidations were present (Figure 2B). General surgery and gastroenterology were consulted; however, the patient was not a candidate for procedural interventions given his advanced metastatic disease. The patient was admitted to the medical intensive care unit and continued receiving broad-spectrum antibiotics. On hospital day 1, the patient's blood cultures returned positive for Clostridium perfringens. The patient was transitioned to comfort care on hospital day 2 and passed away on hospital day 4.

## Discussion

POCUS is a valuable tool that allows clinicians to acquire and interpret images in real time, supporting rapid diagnosis and informed clinical decision-making. Image interpretation often relies on fast, intuitive pattern recognition, especially in high-acuity situations. Research suggests that such heuristics are a fundamental part of human decision-making [3]. However, as this case illustrated, pattern recognition alone comes with a trade-off between speed and accuracy. A deliberate and systematic interpretation should take place in addition to the initial impression to ensure accurate image interpretation.

**Figure 3.**
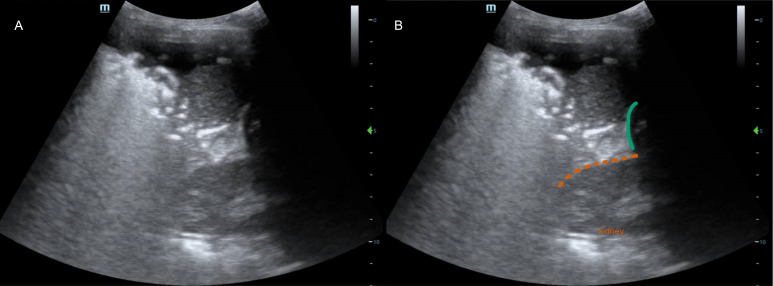
A) Unlabeled point of care ultrasound (POCUS) image of Supplementary Material S1 at 0:02. B) The structures of interest are shown. The kidney, identifiable by its shape and hyperechoic renal sinus, is now visible at a depth of 6 cm (dotted, orange line). The kidney is seen cephalad to the curved hyperechoic line initially interpreted as the diaphragm (green line), demonstrating that the line is not the diaphragm. As such, the structures seen are in the abdomen rather than the thorax.

In this case, the initial appearance of a hyperechoic line with adjacent air bronchograms and a shred sign was misinterpreted as the diaphragm with adjacent lung consolidation. On review, a kidney was seen deep to the curved hyperechoic line, necessitating reinterpretation of the superficial soft-tissue density as the liver instead of a lung consolidation (Figure 3). Thus, the air bronchograms and shred sign seen on POCUS were generated not by a consolidation in the lung, but by air in the liver.

POCUS patterns represent radiographic syndromes rather than specific disease entities. Common lung POCUS syndromes such as air bronchograms and shred sign are created by the interface between air and surrounding soft tissue. Differences in acoustic impedance generate these artifacts [4]. Large air pockets can produce hyperechoic lines with distal reverberation artifacts. The shred sign is an example of this, because it is generated by the interface between consolidated lung and aerated lung in a non-translobar consolidation [1]. In contrast, smaller air bubbles may create bright, punctate foci within hypoechoic structures, as seen with air bronchograms [2]. Any pathology that creates interfaces between air and soft tissue can generate these POCUS signs. In this case, the presence of air in the liver and the portal vein resulted in the shred sign and air bronchograms seen in the abdominal space.

Hoffmann et al. proposed a classification system for abnormal abdominal air based on its location. The categories include extraluminal air, intraluminal air, intraparenchymal air, and intramural air [5]. This framework helps contextualize our patient's findings as a combination of PVG (intraluminal) and air within the hepatic parenchyma (intraparenchymal).

Supplementary Material S1 demonstrates hyperechoic, non-shadowing speckles arranged in a linear pattern, consistent with the appearance of intraluminal air. In the upper right quadrant, this finding is suggestive of pneumobilia or PVG. These pathologies can be difficult to distinguish on ultrasound. Air is typically more centrally located in pneumobilia and peripherally located in PVG [3]. Our POCUS findings most closely align with PVG due to the presence of peripherally located air. M-mode and pulsed wave Doppler can also be used to distinguish PVG. In M-mode, the line of interrogation is placed through the portal vein to track the movement of hyperechoic air bubbles, which resemble a “meteor shower” [6,7]. In pulsed wave Doppler, the sample gate is placed within the portal vein. As air bubbles flow through the sample gate, they generate a hyperechoic vertical spike on the spectral tracing [6,7]. In addition to intraluminal air, Supplementary Material S1 also demonstrates hyperechoic lines with ring-down artifacts within the liver parenchyma, suggestive of intraparenchymal air. This appearance likely represents pyogenic liver abscesses or a necrotic air-filled tumor [8,9]. There are few reports in the POCUS literature on the appearance of air in the abdomen, and even fewer still that describe air so extensive that it produces POCUS signs closely associated with lung POCUS, but in the abdomen.

This case highlights a high-acuity scenario in which using only heuristics in POCUS image interpretation resulted in a significant diagnostic error. The initial recognition of shred sign and air bronchograms without assessment of the surrounding structures led to the incorrect diagnosis of septic shock from pneumonia. A more deliberate review of the images allowed for identification of all the surrounding structures, including the kidneys, which suggested that the pathology was within the abdomen rather than the thorax.

## Conclusion

We report a case of significant abdominal air that produced POCUS images commonly associated with lung POCUS, resulting in misdiagnosis. This case has several major learning points. Firstly, familiarity with the appearance of air in the abdomen on POCUS imaging is crucial in recognizing intra-abdominal pathology. Secondly, common POCUS signs are created by sonographic phenomena and are not pathognomonic for specific pathologies. Finally, clinicians should be cognizant of the role heuristics play in medical judgement, including POCUS image interpretation, and maintain the cognitive flexibility to employ a slower, analytical approach to image interpretation.
